# What Is the Association between Absolute Child Poverty, Poor Governance, and Natural Disasters? A Global Comparison of Some of the Realities of Climate Change

**DOI:** 10.1371/journal.pone.0153296

**Published:** 2016-04-14

**Authors:** Adel Daoud, Björn Halleröd, Debarati Guha-Sapir

**Affiliations:** 1 Visiting Scholar, Department of Sociology, University of Cambridge, Cambridge, United Kingdom; 2 Department of Sociology and Work Science, University of Gothenburg, Gothenburg, Sweden; 3 Centre for Research on the Epidemiology of Disasters (CRED) and University of Louvain School of Public Health, Brussels, Belgium; Hunter College, UNITED STATES

## Abstract

The paper explores the degree to which exposure to *natural disasters* and *poor governance* (quality of governance) is associated with absolute child poverty in sixty-seven middle- and low-income countries. The data is representative for about 2.8 billion of the world´s population. Institutionalist tend to argue that many of society’s ills, including poverty, derive from fragile or inefficient institutions. However, our findings show that although increasing quality of government tends to be associated with less poverty, the negative effects of natural disasters on child poverty are independent of a country´s institutional efficiency. Increasing disaster victims (killed and affected) is associated with higher rates of child poverty. A child´s estimated odds ratio to be in a state of absolute poverty increases by about a factor of 5.7 [95% CI: 1.7 to 18.7] when the average yearly toll of disasters in the child´s country increases by one on a log-10 scale. Better governance correlates with less child poverty, but it does not modify the correlation between child poverty and natural disasters. The results are based on hierarchical regression models that partition the variance into three parts: child, household, and country. The models were cross-sectional and based on observational data from the Demographic Health Survey and the Multiple Indicator Cluster Survey, which were collected at the beginning of the twenty-first millennium. The Sustainable Development Goals are a principle declaration to halt climate change, but they lack a clear plan on how the burden of this change should be shared by the global community. Based on our results, we suggest that the development agencies should take this into account and to articulate more equitable global policies to protect the most vulnerable, specifically children.

## Introduction

The Sustainable Development Goals (SDG) focus more clearly on climate change than the Millennium Development Goals (MDG). While the earlier goal did mention environmental sustainability, the current goals acknowledge the reality of climate change, and that urgent actions need to be taken as this change brings about intensifying frequency of natural disasters and thus human suffering. Scholars have found that people living in rich countries are less affected by disasters, even though the economic damage can be considerable [1, 2]. Besides geographical factors, it has been shown that the adverse effects of disasters hinges on the government capacity to act pro-actively by building a strong infrastructure, installing early-warning systems, and providing a well-equipped health care system, [3–7]. To date, however, little work has been done to link the effects of natural disasters and good governance to child poverty in Middle and Low-income countries. As a result, we still know little about how escalating natural disasters impact this vulnerable group around the world. This is, in turn, hampering the policy makers’ ability to devise effective strategies to protect children. This paper intends to fill some of this gap.

The aim of this paper is to assess to what degree exposure to *natural disasters* is associated with absolute child poverty across sixty-seven low- and middle-income countries (LMICs), and how well good governance moderates this exposure. We translate governmental preparedness into the well-known concept of *quality of governance* (QoG), that is, the administrative effectiveness of a state’s capacity to act effectively [8]. We test how QoG fares in protecting people against the adverse effects of disasters. Our basic assumption is that natural disaster increases the risk that children will become deprived of basic necessities such as food, sanitation, and healthcare, and as a consequence increase their risk of absolute poverty. The assumed mechanisms in play are, first, that QoG has an impact on the direct effects of the disaster and, second, that the recovery phase is shorter and progresses more smoothly in relatively well-governed countries, as compared to ill-governed ones. Consequently, the adverse effects of disasters will cascade into fewer resources to protect children from poverty[9–11], and higher uncertainty in decision making [12].

The analysis is a significant contribution to the research laying at the intersection of global social policy, governance, and climate change. It contributes to the general discussion about why peoples’ living conditions vary between countries and, more specifically, to the increasing interest in understanding the detrimental effects of bad governance.

One additional novelty of the current paper is its research design. It is unique because it combines country-level data about disasters with microdata about children’s exposure to deprivation across a large number of LMICs: representative of about 2.8 billion of the world´s population.

Previous research has either had a longitudinal design focusing on the country level and therefore not being able to include microdata on the child level [3]; or, it has focused on the household level and not incorporated country level data [13]. Our research incorporates some of the strengths of combining both macro and micro level data. It focuses on between-country variations since the data on disasters is collected at the country level; the study has a cross-sectional design since we currently only have cross-sectional data on poverty. We will be addressing these limitations in future research.

In the following section, we discuss some of the key findings on the relationship between natural disasters, institutional conditions, and child poverty. We outline the underlying theoretical model of this paper. In section three, we discuss data collection both on the macro level (natural disasters, institutions, and alike) and micro level (child poverty data) used in this study. In section four, we outline the results. We conclude by discussing our interpretation of the results and offering further recommendations for policy and future research.

## Background: Natural Disasters, Institutions, and Child Poverty

The idea that natural disasters lead to poverty and possibly to mass death are—or, at least, has been—central to some areas of development studies; its roots can be found in basic Malthusian concepts ([14], cf. [15, 16]). In contemporary research, it is clear that a natural disaster has a social, economic, and natural component [1, 7, 17, 18]. It is not purely *natural*, and thus its adverse effects can be counteracted by technological and institutional mechanisms (cf. [4]). A natural hazard becomes a disaster when it interacts with human beings in a negative way: for example, earthquakes occur in many places, but an earthquake only becomes a disaster when people are affected by it and if they do not have the means to protect themselves (resilience). Lal et al. make the causal and conceptual distinction between “natural disasters leading to poverty,” on the one hand, and “poverty [that] may lead to increased vulnerability to natural hazards,” on the other [19, 20]. Both causal directions are viable. With this background, and because of data limitations, this study will mainly focus on exploring the association between natural disasters, governance, and poverty, rather than seeking to determine causal connections.

We still expect that more disasters are correlated with higher proportions of poverty: child poverty, specifically (cf. [21, 22, 23]). Our framework assumes that the first line of defense against the adverse effect of a disaster is the household determined by the capabilities of the adults [19, 24, 25]. The second line of defense is the protective measures that governments take to empower households and to set up the necessary infrastructure. Most research seems to focus on the role that governments play in preparing for disasters and in organizing help when disasters occur (e.g. [5, 6, 23, 26]). Strömberg [3], for example, shows that both developed countries and LMICs are affected by natural disasters almost to the same degree, but still people are injured or killed to a far less extent in developed countries where the quality of government and economic development is higher than in LMIC. We know, moreover, that QoG explains country differences in child deprivation [27, 28]. These observations warrant our research question to analyse how the level of the quality of government moderates the disaster effect on child poverty.

Our account rests on the following logic: If the QoG effects disappear once disasters are part of the equation, we can conclude that earlier research results on QoG might have been based on spurious observations. If QoG wipes out the impact of disasters, we can conclude that it is QoG that matters for child deprivation. Hence, natural disasters affect children either by harming the directly, killing or severely harming their parents, or by destroying property. Effective (good) governments can influence the effect of disasters on family livelihood expenses in at least two ways: they can reduce the potential damage of future disasters by enforcing certain policies (e.g., anti-earthquake building policies), and they can moderate the effects of disasters, for example, by providing financial support to households and by other economic policies that re-establish the functioning of the economy (e.g., repair of the infrastructure).

## Methods

### Microdata on Absolute Child Poverty and Severe Child Deprivation

Our individual-level data on child deprivation come from the Demographic and Health Survey (DHS) organized by US-AID and UNICEF’s Multiple Indicator Cluster Survey (MICS), two well-established household survey programs. UNICEF works in close collaboration with the DHS program to harmonize survey questions and modules and to ensure a coordinated approach to survey implementation. These surveys are nationally representative with large sample sizes, high quality, and high response rates [29]. The DHS has been conducted since 1984 and is now running the sixth wave of surveys. UNICEF initiated the Multiple Indicator Cluster Surveys (MICS) in the 1990s and is now conducting the fifth wave of surveys. To date, over 350 DHS and MICS have been carried out in 115 LMICs. This study includes 67 countries, with surveys collected by the DHS and MICS around the year 2000, with a variation of about seven years. These countries represent the bulk of low and middle-income countries in the world. The survey selection for this analysis was determined by what data we have harmonized at the time of writing, which included surveys mainly from MICS first and second round as well as DHS round three plus four. We are in the process of conducting further data harmonization and will thus be able to extend this analysis.

The literature on the measurement of poverty and child deprivation is rich: household income based methods, most notably the World Bank dollar a day method; non-income based measures, such as: under-five mortality, primary school enrolment, malnutrition (wasting, stunting, underweight), and access to improved sanitation or water facilities [30]. Some of the advantages of the deprivation based approaches are that they will direct our attention to what matters, that is, whether the child is well-nourished, healthy, and educated: which in turn lays the foundation for this child to develop and flourish freely [31].

Alkire and Foster´s Multidimensional Index from the Oxford Poverty and Human Development Initiative [32] and the Bristol method are the two most well-known deprivation based approaches to child poverty. They are also amenable to quantitative analysis [33] One of the main limitation of the Multidimensional Index is that it does not measure poverty on the child level but rather at the household level [34]. Even if household approaches adjust for household composition, they do not easily lend themselves to a within-household analysis. While the Bristol method has its limitations [35] it provides one of the most versatile approaches to measuring child poverty. A significant strength of the Bristol Method is that it is based on internationally agreed definitions of poverty. For this reason, UNICEF used it to assess absolute poverty among children around the world [36]. The approach implements the internationally accepted definition of poverty—adopted at the 1995 World Summit on Social Development in Copenhagen, which states that absolute child poverty is:

"…a condition characterised by severe deprivation of basic human needs, including food, safe drinking water, sanitation facilities, health, shelter, education and information. It depends not only on income but also on access to services."

[37]

Hence, we focus on the Bristol approach in this study [34, 38]. With a basis in the human rights approach derived from the 1995 World Summit, this approach operationalizes the seven deprivations types. The following three deprivation types apply to children under 18 years old. (1) *Water*: Children who only had access to surface water (for example, rivers) for drinking or who lived in households where the nearest source of water was more than 15 minutes away. (2) *Shelter*: Children in dwellings with more than five people per room and/or with no flooring material. (3) *Sanitation*: Children who had no access to a toilet of any kind in the vicinity of their dwelling, that is, no private or communal toilets or latrines. (4) *Food*: Children whose heights and weights for their age were more than -3 standard deviations below the median of the international reference, that is, severe anthropometric failure. This deprivation type applies to children under five years old only. (5) *Health*: Children who had not been immunized against diseases or young children who had a recent illness involving diarrhea and had not received any medical advice or treatment. This deprivation type also applies only to children under five years old. (6) *Education*: Children who had never been to school and were not currently attending school, i.e., no professional education of any kind. Education is measured for children 7 to 12 years old. (7) *Information*: Children who had no access to radio, television, telephone or newspapers at home. Applies to children with the age 3 to 12 years. According to the definitions outlined in the World Summit, a child is in a state of *absolute child poverty* if that child has two or more deprivations of the seven outcomes.

Table 1 outlines the 67 countries included in the study. Our combined sample contains information on 1,941,734 children under the age of 18 and for 567,344 households.

**Table 1:** Countries in the study, sample year and sample size.

Country	Year	n
**Albania**	2000	6683
**Angola**	2001	16535
**Azerbaijan**	2000	9732
**Bangladesh**	2006	127250
**Armenia**	2000	8300
**Bolivia**	2004	37856
**Bosnia and Herzegovina**	2000	9486
**Brazil**	1996	22732
**Burundi**	2005	22799
**Cambodia**	2006	33463
**Cameroon**	2006	21303
**Chad**	2000	16209
**Colombia**	2005	60392
**Congo BR**	2005	14526
**Congo, DR**	2007	25219
**Benin**	2001	16215
**Dominican Republic**	2002	47253
**Ethiopia**	1992	34439
**Gabon**	2000	15687
**Gambia**	2000	14191
**Ghana**	2006	12742
**Guatemala**	1999	16239
**Guinea**	1999	18745
**Guyana**	2001	8733
**Haiti**	2000	22983
**India**	2006	198294
**Indonesia**	2003	56726
**Iraq**	2000	50775
**Jamaica**	2005	5813
**Jordan**	1997	22114
**Kenya**	2003	18779
**Kyrgyzstan**	2006	10015
**Laos**	2006	16263
**Lesotho**	2000	14352
**Madagascar**	2004	36625
**Malawi**	2006	71425
**Mali**	2006	40095
**Mongolia**	2000	11576
**Morocco**	2004	24439
**Mozambique**	1997	23245
**Namibia**	2000	15031
**Nepal**	2006	19935
**Nicaragua**	2001	29673
**Niger**	2006	27180
**Nigeria**	2007	63188
**Pakistan**	2007	32994
**Peru**	2004	11040
**Philippines**	2003	26768
**Rwanda**	2000	24911
**Senegal**	2005	35446
**Sierra Leone**	2006	21022
**Vietnam**	2006	12736
**South Africa**	1998	24019
**Zimbabwe**	2006	21218
**Sudan**	2000	81451
**Suriname**	2000	6603
**Swaziland**	2000	12557
**Tajikistan**	2000	12711
**Thailand**	2006	38954
**Uganda**	2001	21448
**Ukraine**	2005	5830
**Egypt**	2005	45155
**Tanzania**	2005	25022
**Burkina Faso**	2003	32831
**Uzbekistan**	2006	19906
**Yemen**	2006	13637
**Zambia**	2002	20220

### Measuring Climate Change via Natural Disasters

Data on natural disasters have been collected from the Emergency Events Database (EM-DAT), which is maintained by the Centre for Research on the Epidemiology of Disasters (CRED) at the University of Louvain. For a discussion of data problems and a comparison of available data sources, see Kron et al. [39]. EM-DAT contains core data on the occurrence and effects of more than 17,000 disasters in the world from 1900 to the present (our analysis stretches to the year 2012). It contains, for example, data on the type of disaster, the number of people killed and injured, and the number affected. For a disaster to be entered into the database, at least, one of the following criteria must be fulfilled: ten or more people were reported killed; a hundred or more people were reported affected; a state of emergency was declared, or a call for international assistance was issued. We chose 1988 as the starting date for our sample since this is the year CRED created the EM-DAT and thus started to collect disaster data in a more systematic manner. One would, therefore, expect that the monitoring of disasters, data collection, and validation to be of higher quality than disaster data before this date [40].

**Fig 1:**
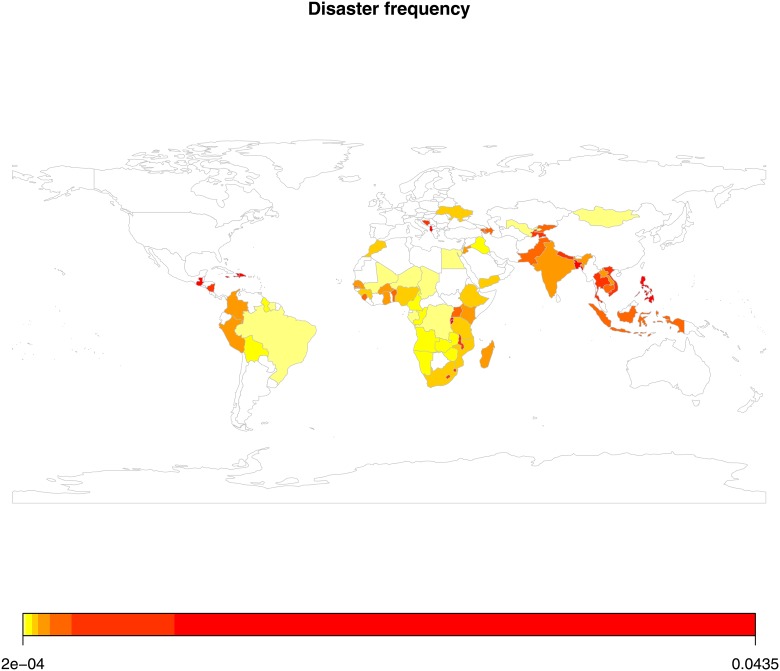
Disaster frequency in sample countries across the globe. *Notes*: (a) Authors graph calculated from EM-DAT. (b) the red colour depicts a higher average intensity of disaster frequency.

**Fig 2:**
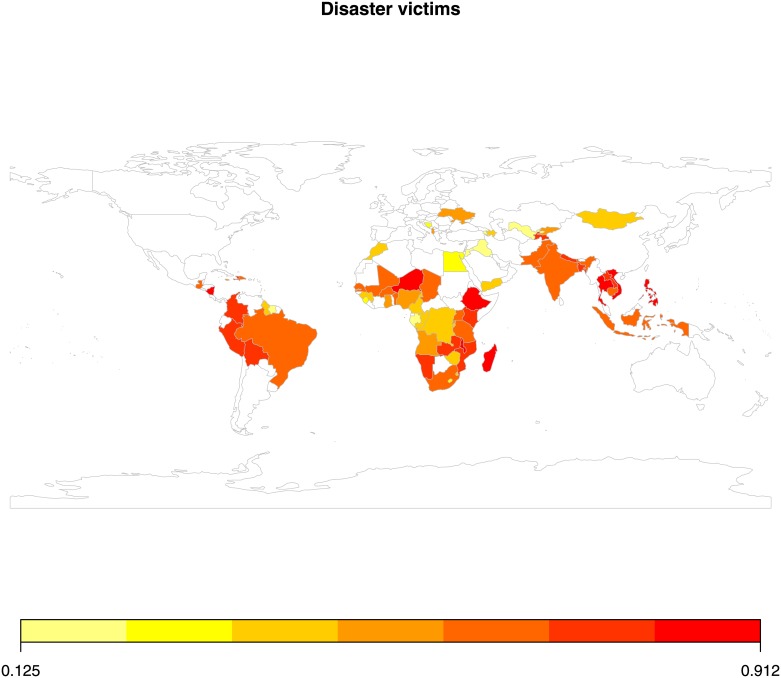
Disaster victims in sample countries across the globe. *Notes*: (a) Authors graph calculated from EM-DAT. (b) the red colour depicts a higher average intensity of disaster victims.

Since we are mainly interested in the average tendency of natural disasters, we have calculated two kinds of yearly average for the defined twenty-five-year interval covering 1988 to 2012. A country with a frequency of ten earthquakes during this twenty-five year period will have an earthquake rate, ten divided by twenty-five, which is 0.4. The same logic applies to the number of people affected. If earthquakes affected 25,000 individuals within a country during the period of our study, the country would have an average of thousand individuals affected per year. As a result, we constructed two types of measures: one capturing the average occurrence or disaster frequency that a country has had during our observation period and another one capturing the average number of disaster victims, i.e., those killed and otherwise affected during the same period. Since we are comparing countries that differ when it comes to both geographical and population size, we also need to adjust for country size. So, for the models involving disaster frequency we have included the countries’ geographical size, and for the disaster victims, we have controlled for the countries’ population size. Figs 1 and 2 give a graphical representation of the distribution of values in the sample across the globe.

The paper focuses on analyzing the disaster frequency and victims measures. They are aggregates of four different types of disasters (www.emdat.be). Climatological disasters, “Events caused by long-lived/meso- to macro-scale processes (in the spectrum from intraseasonal to multidecadal climate variability)”, for example, drought and wildfire. Geophysical disasters, “Events originating from solid earth”, such as earthquakes and volcanos. Hydrological disasters, “Events caused by deviations in the normal water cycle and/or overflow of bodies of water caused by wind set-up”, most notably, floods. Plus, meteorological disasters, “Events caused by short-lived/small to mesoscale atmospheric processes (in the spectrum from minutes to days), such as storms”. We omitted the category biological disaster since its classification as a *natural* disaster is not entirely solid. Of these four disasters categories, it is only geophysical disasters (earthquakes, volcanos, mass movement (dry)) that might have little to do with climate change. The other three, meteorological (storms), hydrological (floods), and climatological (droughts, extreme temperature and wildfire) are strongly related to it. However, geophysical disasters do makeup only a small portion of the total and correlate highly (about 0.9) with the aggregate measure. To capture mass movement (dry) as well, we decided to use the full category of geophysical disasters for the sake of consistency.

The disaster variables were transformed using the 10-base logarithm to force the data to have a smaller spread and avoid extreme cases. First the raw unadjusted disaster figures were calculated (for frequency and victims); Then the 10-base logarithm used to make yet another transformation. The distribution is still skewed towards zero, which is the result of a Poisson like process that disasters tend to have. We tested other transformations, but we settled for the 10-base logarithm since its interpretation is more intuitive and has also been used in such classical work as in Richardson [41].

### Measuring Quality of Governance

Other country-level data have been collected from the *Quality of Governance* database, QoG Institute, University of Gothenburg. The QoG database covers 194 countries and is one of the most complete databases on social policy and the quality of government, which is essentially a result of data harvesting from sources such as the World Bank, the UN, and its sub-organizations [42]. To measure the quality of government (QoG) we use the government effectiveness estimate (wbgi_gee) of Kaufmann, Kraay, and Mastruzzi [43, 44]. The measure is based on a compilation of information from survey data and expert assessments supplied by a range of organizations. In total, 36 components have been gathered from 15 different organizations, which “combines into a single grouping responses on the quality of public service provision, the quality of the bureaucracy, the competence of civil servants, the independence of the civil service from political pressures, and the credibility of the government’s commitment to policies. The main focus of this index is on ‘inputs’ required for the government to be able to produce and implement sound policies and deliver public goods” [45]. This measure comes as a standardized measure in which the mean is zero, and the standard deviation is set to one. (See Arndt [46] or Agnafor [47] for a critical review of available governance measures.).

In this study, we have taken the historical average, 1988 to 2012, for each and every country as a new proxy for the quality of government since we have a corresponding historical average for the natural disaster variables. Note that Kaufman and his colleagues standardize the measure with a mean of zero, which means that the historical average we are using is based on the relative ranking of the country. The countries included in this study are mainly found in the below the average of the global QoG distribution.

### Additional Control Variables

We controlled for confounders by including a set of additional covariates. At the country level, we add a measure of democratization controlling for the assumption that, in democracies, politicians tend to implement policies improving the living conditions even for the poor because they are held accountable in free and open elections [48]. In an assessment of alternative indices of democracy, Hadenius and Teorell conclude that the measures provided by Freedom House and Polity IV Project provide the most valid and consistent albeit not perfect measures of the procedural aspects of democratization [45]. They further suggest that a combination of these two measures produces the most preferable measure: achieved by taking the average of the Freedom House and transforming it to a scale 0–10 and Polity to the same range, and then averaging the two variable into a new democracy measure (. It is this combination we use as our indicator of democratization. The new scale also ranges from 0 to 10, in which 0 is least democratic and 10 most democratic. Also, since the occurrence of natural disasters varies between years, we also include a survey year as a control variable. At the household and child level, we control for the household composition, measuring the number of children and adults. We also control for the children’s age and sex. Finally, since child poverty still is a predominantly rural phenomenon [27], we include a variable that measures whether children live in urban or rural areas. All the sources of the country-level measures used in this study are outlined in Table 2.

**Table 2:** Country level variables used in the study and their sources.

	median	mean	std.dev	sources
Disaster frequency	0.00	0.00	0.01	Centre for Research on the Epidemiology of Disasters
Disaster victims	0.60	0.56	0.21	Centre for Research on the Epidemiology of Disasters
Democracy	4.60	4.83	2.23	Freedom House and Polity IV Project
GDP	5.77	5.78	0.36	World Development Indicators
Population (thousands)	10820.58	41366.81	121505.98	World Development Indicators
Country area (thousands)	330615.71	700862.79	1169837.96	World Development Indicators

Note: All country level variables used in the study. They are all historical averages, 1988–2012.

To match the averaging procedure we applied to the disasters measures and the QoG measure, all the country level variables have been averaged for the period 1988 to 2012.

### Statistical Analysis

Given our theoretical framework, we specified three baseline models. All models are logistic since the outcome variable is a binary measure (poor or not poor). The first baseline model Eq (m1) tests the hypothesis that there is an adverse effect of disasters on child poverty without any moderation of QoG, but controlled for it. This model including a set of other covariates to control for confounders: hence, *Probability*(poverty = 1|Disasters, QoG,…Covriates). To reiterate, we measured natural disasters both as the average yearly disaster victims and the average yearly disaster frequencies. We expect that higher disaster rates should correlate positively with higher deprivation rates, i.e., we expect *β*_1_ to be positive. We still relied on a two-tailed interpretation of the relevant coefficients, to make the interpretation more conservative.

$$logit(\;y_{\left(d\right)\text{ijk}})=\;\beta_{0}+\;\beta_{1}X_{Dis}+\;\beta_{2}X_{QoG}+\;\beta_{4}X_{controls}+\;c_{0k}+\;h_{0jk}+\;\varepsilon_{ijk}$$ (m1)

$$logit(\;y_{\left(d\right)\text{ijk}})=\;\beta_{0}+\;\beta_{1}X_{Dis}+\;\beta_{2}X_{QoG}+\beta_{3}X_{QoG}*X_{Dis}\;+\;\beta_{4}X_{controls}+\;c_{0k}+\;h_{0jk}+\;\varepsilon_{ijk}\;+$$ (m2)

$$logit(\;y_{\left(d\right)\text{ijk}})=\;\beta_{0}+\;\beta_{1}X_{DisFre}+\;\beta_{2}X_{QoG}+\beta_{3}X_{DisVic}+\beta_{4}X_{QoG}*X_{DisFre}+\;\beta_{5}X_{QoG}*X_{DisVic}+\beta_{6}X_{DisFre}*X_{DisVic}+\beta_{7}X_{QoG}*X_{DisFre}*X_{DisVic}+\beta_{8}X_{controls}+\;c_{0k}+\;h_{0jk}+\;\varepsilon_{ijk}$$ (m3)

*All models are logistic random intercept models with three random terms*: *one term for the child level ε*_*ijk*_ *(child i in household j*, *in country k)*, *one for the household h*_0jk_, *and one for the country level c*_0k_ *for the jth country*.

Indices:

***d*** = deprivation measure, where *d* ∈ [absolute child poverty]
***Dis*** = disaster measure, where *Dis* ∈ [disaster frequency (DisFre), disaster victims(DisVic)]
***QoG*** = Quality of Government measure
***Controls*** = [the child´s age, nr of children in the household, nr of adults in the household, the child´s sex, household location, democracy, GDP, population size, country size]

The second baseline model Eq (m2) tests the hypothesis that the association between disasters and child poverty is conditioned (moderated) by the level of QoG. We still assume that the coefficient (*β*_1_) is positive, for the disasters parameter but that the interaction parameter (*β*_3_) should damper this effect. In other words, higher levels of QoG (better governance) are assumed to decrease the adverse effect of disasters.

We also defined a third baseline model Eq (m3): partially for the sake of further exploration, partially for the sake of robustness. This model tests if the effect of disaster victims depends on both the levels of disaster frequency and the level of QoG. In that model, we assume that the disaster parameters to be negative (*β*_1_ and *β*_3_) and their corresponding interaction parameters to yield a negative marginal effect. We assume that the higher the rate of frequency is, the more pronounced the effect of disaster victims is on child poverty. Moreover, our theoretical framework states that this effect should be dampened by higher (better governance quality) rates of QoG.

We modelled these three baseline models in the following way. First we fitted a null model to identify the variance across the defined levels (country, household, and child) that are captured by the corresponding residuals (***c***_**0k**_,***h***_**0jk**_, and ***ε***_***ijk***_). This gave us the amount of variance that country level variables can explain at most. Then we modelled the simple bivariate relationship between the disaster victims and absolute child poverty. This revealed if there are any bivariate associations between these two focal events. After that we added all the controls to see if this focal relationship remained robust. We considered this step to be the main test of the first hypothesis—concerning disasters evens as captured by disaster victims—which is captured by Eq m1. Finally, we also tested our second hypothesis by adding the interaction terms as defined by Eq m2. We repeated the same process but exchanged disaster victims for disaster frequency to test the analogous hypotheses but now operationalized as the average yearly frequency of disasters. After following these seven modelling steps, we tested the Eq m3 formulation. Lastly, we also removed QoG entirely from Eq m3 in case the focal association only depended on the two disasters measures: frequencies and victimization processes. Hence, this analysis sums up to nine models in total.

In both linear and non-linear models, when one merely estimates *β*_3_ one is, strictly speaking, not testing the full conditional effect of disasters on poverty moderated by QoG, but merely an existence of some moderation [49–51]. If we take Eq m2 and if we assume it was linear, where *β*_4_*X*_*controls*_ designates a set of control variables: *y*_(*d*)_ = *β*_0_+*β*_1_*X*_*QoG*_+*β*_2_*X*_*Dis*_+*β*_3_*X*_*QoG*_*X*_*Dis*_+*β*_4_*X*_*controls*_, then the marginal effect of disasters on deprivation is then acquired by a differentiation with regard to disasters, which yields $\frac{\partial \;y_{\left(d\right)\;}}{\partial \;X_{Dis}}\;=\;\beta_{2}+\beta_{3}X_{QoG\;}$. Likewise, the marginal effect of QoG on poverty moderated by disasters is $\frac{\partial \;y_{\left(d\right)}}{\partial \;X_{QoG\;}}\;=\;\beta_{1}+\beta_{3}X_{Dis\;}$. In non-linear models, as the ones we have defined here, this differentiation has to be done with respect to the logit function and which gives a more complicated term. The marginal effect of disasters is now: $\frac{\partial \;y_{\left(d\right)}}{\partial \;X_{Dis\;}}\;=\;p*\left(1-p\right)*(\beta_{1}+\beta_{3}X_{QoG\;}),$ where *p* = *Logit*(Poverty = 1 | *X*_*Dis*_,*X*_*QoG*_ …*X*_*controls*_). What can be determined from a t-test of *β*_3_ is whether a conditional relationship is present. Therefore, in the case of Eq m2, a simple t-test will suffice when testing for moderation effect [51]. But one will not know from this simple test for which subset of QoG values this moderation is statistically significant. We will thus (a) check if a conditional interaction exists between disasters and QoG by conducting a simple t-test of the coefficient of interaction term (*β*_3_), and (b) if a relevant effect exist, we will then go on to investigate this interaction further with the marginal effect.

In the case of Eq m3, a three-way interaction, the relationship between the parameters follows the same principles as they do in Eq m2, even if the expression is a bit more complex. Similarly, we will focus on interpreting *β*_6_,

This study utilizes multilevel statistical modeling [52, 53]. Such techniques capture the partitioning of variance for the different nested levels and thus more clearly highlight where the strongest associations exist between variables. All the models are multilevel models with three levels since there is a significant amount of variation partitioned on each level (children, households, and countries). Moreover, our dependent variable is defined at the micro-level, and our focal independent variable is defined at the macro-level. In such a design, we are testing between-country variation rather than within-country: we cannot test how disasters within a country affect the population in various ways. What we are testing is how child poverty in a country, on average, is associated with that country’s level of disasters, on average. Our models (between disasters and poverty) do not commit an ecological fallacy: that is, generalizing group (country)-level characteristics to the individual (child) level. Statistically, the reason for this is simple; our country-level variables can only explain variation that exists at the country level (aggregated from the child-level dependent variable) [54].

To reiterate, because our microdata does not allow for a pre- and post- natural disasters effect analysis, our research design is cross-sectional. For that reason, and to align all the macro level measures to the historical averaged natural disaster measures, we conduct the similar averaging procedure of QoG, economic development, and democracy. This averaging procedure implies that we are less sensitive to the temporal relationship between the defined macro variables—and to the initial condition problem [55].

We want to emphasize again that the main focus of this paper is to investigate if there is a unique effect of disasters on absolute poverty potentially conditioned by QoG and not to explain as much variation in the outcome variable. In other words, we are not maximizing the coefficient of determination (R squared) with as many covariates as possible, but rather analyzing the unique effect of the disaster parameters [56]. Within this agenda, we will use the deviance information criterion (DIC) to differentiate between the various models we have fitted.

All models were estimated using the MLwiN engine, using the Markov Chain Monte Carlo (MCMC) estimation algorithm [57]. We used the R2MLwinN package [57] in the R software [58] to call MLwiN and thus to manage as well as to analyze the results.

## Results

Table 3 outlines all the regression models. Model 1 is a null model with no covariates defined. This model shows that most of the variance in absolute child poverty reside between countries: 49 percent. The variation between households is 39 percent, and the rest is between children.

**Table 3:** The effect of disasters and governance on absolute child poverty: Odds ratios.

	Model 1	Model 2	Model 3	Model 4	Model 5	Model 6	Model 7	Model 8	Model 9
Intercept	0.13***	0.00***	16.26	16.20	0.12***	8611.16	1280.22	305.83	1732.35
	(0.06)	(0.00)	(133.59)	(137.51)	(0.06)	(57773.23)	(9000.94)	(3480.27)	(14886.04)
Household and Child variables age									
			0.98***	0.98***		0.98***	0.98***	0.98***	0.98***
			(0.00)	(0.00)		(0.00)	(0.00)	(0.00)	(0.00)
nrchildren			1.06***	1.06***		1.06***	1.06***	1.06***	1.06***
			(0.00)	(0.00)		(0.00)	(0.00)	(0.00)	(0.00)
nradults			0.90***	0.90***		0.90***	0.90***	0.90***	0.90***
			(0.00)	(0.00)		(0.00)	(0.00)	(0.00)	(0.00)
Boy (ref = girl)			0.99^·^	0.99^·^		0.99^·^	0.99^·^	0.99^·^	0.99^·^
			(0.01)	(0.01)		(0.01)	(0.01)	(0.01)	(0.01)
Urban (ref = rural)			34.24***	34.22***		34.23***	34.24***	34.20***	34.27***
			(0.38)	(0.39)		(0.42)	(0.40)	(0.40)	(0.40)
Country variables									
Democracy			0.97	0.97		1.02	0.99	0.80	0.81
			(0.19)	(0.19)		(0.20)	(0.20)	(0.17)	(0.16)
GDP			0.10*	0.10*		0.02***	0.03**	0.05**	0.04**
			(0.11)	(0.11)		(0.03)	(0.04)	(0.05)	(0.05)
QoG			0.12*	0.15		0.25	0.27	0.09	0.24
			(0.13)	(0.71)		(0.25)	(0.27)	(0.61)	(0.23)
Population size			0.51	0.51				0.04**	0.04**
			(0.32)	(0.35)				(0.04)	(0.04)
DisVic		6.26**	5.66**	5.49^·^				5.12	3.88*
		(3.94)	(3.46)	(5.51)				(6.89)	(2.46)
DisVic:QoG				0.96				1.35	
				(0.94)				(2.04)	
DisFre					3.63	1.31	0.56	12.88	1.94
					(3.91)	(1.17)	(0.70)	(78.22)	(7.88)
Country size						4.17*	4.61**	12.24***	11.47***
						(2.40)	(2.73)	(7.99)	(7.30)
DisFre:QoG							0.14	276.93	
							(0.29)	(3065.84)	
DisFre:DisVic								0.81	1.34
								(0.95)	(1.10)
DisFre:DisVic:QoG								0.25	
								(0.55)	
Country variance	14.68	13.11	7.32	7.41	14.58	7.32	7.34	6.32	6.06
Household variance	11.52	11.53	6.57	6.57	11.52	6.57	6.57	6.57	6.58
Child variance	3.29	3.29	3.29	3.29	3.29	3.29	3.29	3.29	3.29
Countries Num. obs.	67.00	67.00	67.00	67.00	67.00	67.00	67.00	67.00	67.00
Households Num. obs.	567344	567344	567344	567344	567344	567344	567344	567344	567344
Children Num. obs.	1941734	1941734	1941581	1941581	1941734	1941581	1941581	1941581	1941581
DIC	1358147.88	1357984.25	1342294.25	1342373.62	1358189.62	1342318.25	1342300.88	1342351.88	1342240.38

***p < 0.001,

**p < 0.01,

*p < 0.05,

^**·**^ p < 0.1.

Outcome is absolute child poverty. Logistic model, using MLwiN 2.35 MCMM (15000 Iterations; 5000 burn-in). Odds ratio SE in parentheses. DisFre is disaster frequency. DisVic is disaster victims. QoG is quality of government.

The control covariates—that is, the variables other than disaster victims, disaster frequency and QoG—have expected effects: older children have a lesser odds (a factor of 2 percent less for each year) of being poor; mothers in household with more children are more likely to give birth to children into a state of poverty (6 percent more likely); households with more adults have a protective effect (10 percent less likely); children born up in rural households have a much larger odds ratio of being poor (34.24 times likely). Wealthier countries are also less likely to have children that are poor, and children living in larger countries are more likely to be poor. Population size, the level of democracy, and the sex of a child have no significant effect. We find similar results on the absence of democracy effect in a previous study [27].

Disaster frequency has no statistically significant effect across all its relevant models (models 5 through 7). It does neither carry any own unique effect on absolute child poverty nor does its effect depend on the level of QoG. As a further set of robustness checks for this piece of result, we specified two more models. Model 8 is a three-way interaction between disaster frequency, disaster victims, and QoG. It tests if the effect of disaster victims depends on both the levels of disaster frequency and QoG. Our estimations could not detect such an effect. Model 9 estimates a model where the effects of disaster frequency and disaster victims depends on the level of the other one. Again, the interaction term between disaster frequency disaster victims was not significant. This result indicates that, given our research design, natural disasters as measured by frequency have no effect on child poverty. This could be the case because frequencies do not disentangle the effect of natural hazards (mere occurrence of an event)) and natural disasters (an occurrence that has affected people) properly. This statement gains further support when we analyse the relationship between disaster victims and child poverty.

Model 2 and model 5 show that disaster victims have a significant effect on absolute child poverty. In a bivariate relationship (model 2), the model estimates that for a one-unit increase of disaster victims on a log-10 scale, any child´s odds ratio is factored by 6.26. This ratio is estimated to be somewhat lower in models 3 and 4. This could either be due to true reduction in effect accounted for by confounding variables, or part of the scaling issue of logit models. Model 3 shows that both QoG and disaster victims carry own independent effect on a child poverty. For a one-unit improvement of QoG—which is one standard deviation on the normalized QoG scale (see variable description above)–a child is 10 times less likely to be poor. Model 4 shows that the effect of disaster victims does not depend on any moderation effect of QoG since the interaction terms are not significant. Moreover, the models´ deviance information criterion (DIC) shows that model 3 is superior to model 4. It should be observed that model 9 has the best overall (lowest) DIC. However, given our substantive analyses of models 5 through 8, we suggest that model 3 is the model that accounts for our research question best—and that it is most parsimonious. Therefore, we conclude that disaster victims and QoG carry independent effects on absolute child poverty and that the strength of the disaster victims effect does not depend on the level of QoG.

**Fig 3:**
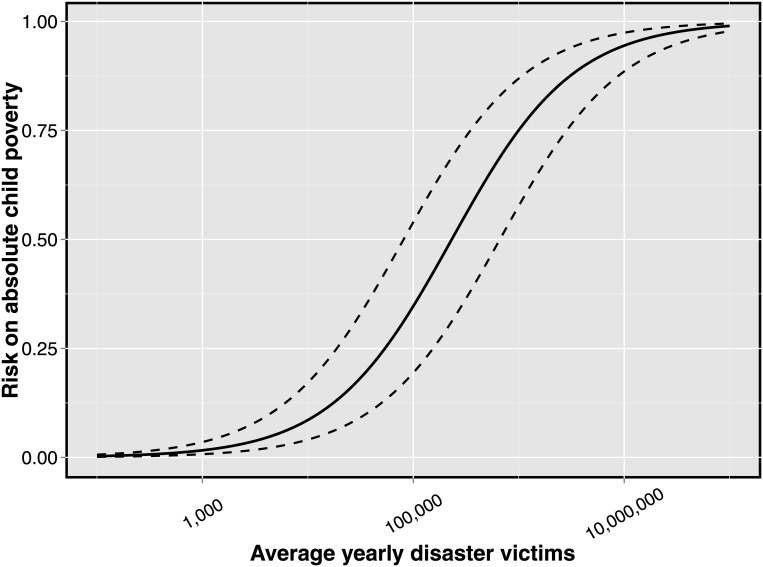
The association between natural disasters and absolute child poverty. *Notes*: (a) Authors calculations based model 3. (b) logarithmic x-scale. (c) Predictions include 90-% confidence interval.

Based on model 3, Fig 3 shows the predicted probabilities of a child´s increasing risk of being poor, when its country´s average yearly disaster victims increases. This prediction holds all relevant covariates at their sample mean. The prediction shows that children living in countries with high rate of disaster victims are strongly associated with them being poor. The top-five-highest-disaster-victims countries in our sample are: India, Kenya, Bangladesh, Ethiopia, and Thailand. In that order. These countries have an average yearly disaster victim rate between about 800 000 victims to 3 100 000 million victims. In that disaster victim range, the risk for a child to being poor, arising purely from natural disasters´ toll is in the probability range 72 to 87 percent. This prediction is, to reiterate, controlled for other risk factors and possible confounders. Hence, in a context of global climate change, the toll of natural disasters on children cannot be ignored.

## Discussion

A central theoretical question of this paper was whether good governance (QoG) could dampen the adverse effects of disasters. There is ample evidence that the lack of QoG has a negative impact on a range of aspects, including health, access to healthcare, education, and mortality [59–63]. We cannot find such a dampening effect. We have shown that countries that have high average rates of natural disaster victims, also create a socio-economic environment where children have a higher risk of finding themselves in a state of absolute poverty. Even if more developed QoG leads to less child poverty, the effect of natural disasters is independent of the level of QoG of the country. Children living in countries with a high disaster victimization rate—e.g. India, Kenya, Bangladesh, Ethiopia, and Thailand—will need more attention to escape poverty. We also note that democratization of a country has no effect on absolute child poverty. Our results suggest that merely improving QoG—or democratization processes—will not help to counter the direct effects climate change. Targeted policy interventions are needed to improve the lives of children around the world.

Before we discuss some policy implications of our findings, it is important to note its limitations. First, in this study we used a cross-sectional approach to analyse the data, which is a rather standard limitation when studying low and middle-income countries involving micro-data. With a longitudinal design on both the macro and micro level, we would be able to set up a quasi-experimental design (e.g. difference-in-difference or regression discontinuity design) and thus say more about causal connections. At the time of conducting this study, we have harmonized cross-sectional micro data spanning around the year 2000, which also comprises our survey selection condition. We are, however, conducting further harmonization of the microdata that would allow for a repeated cross-sectional design, and thus, test for some time-series effects.

Second, we aggregated the disasters to the country level, and that masks an important portion of the total variation: the within-country variation. This type of variation is important to model since it captures how children within the same country are hit differently by natural disasters. For example, a child living near a river that has swollen will be hit directly by that flooded area, compared to a child remotely located. The International Disaster Database currently lack spatial or geographical information system (GIS) data; a large part of the microdata has such data. With such GIS data, we could have deployed more sophisticated methodologies such as hierarchical spatial models [64]. This methodology can locate the area of effect of disasters and its proximity to child poverty data. Statistical tests of this sort would be more reliable. Researchers at the International Disaster Database is currently working to update the database with GIS information.

Third, we rely mainly on the World Bank´s definition of QoG focusing on institutional effectiveness, which is the measure most extensively used in the literature. The debate about what good governance should contain, is extensive [65]. Some argue that governance should be defined as impartiality and absence of corruption [42, 66]. Others show that moral contents are necessary [47]. However, most of these approaches might be theoretically developed, but are not backed with deployable empirical data. This means that some of these theoretically important elements of the good governance concept are not captured in this study. Hence, this also means that any conclusions drawn from this study, apply mainly to the World Bank´s definition of governance.

Notwithstanding these limitations, our design has some advantages. We can cover more countries in the analysis, and combine macro as well as microdata simultaneously. As discussed in the methods section, our statistical technique avoids committing an ecological fallacy by separating out the country level variance from the household, and the child level variance.: not higher level variables can explain any lower level variance. Our disaster data summarizes about 25 years of trends, covering a large portion of the world´s population: about 2.8 billion.

To best of our knowledge, this is the first study of its kind that links natural disasters to absolute child poverty in a global comparison. Our results are also plausible: that natural disasters victimization correlates with increasing rates of child poverty. Previous studies find similar types of associations between natural disasters and public health in general, but in other settings, for example: the impact of earthquakes on the quality of life in Indonesian [67]; the effect of floods on public health in Vietnamese, [13], the links between climate variability with crop production and food security in Tanzania [68], disasters and poverty outcomes in Fiji [20], poverty traps in Ethiopia and Honduras, [69], economic impacts of disaster in Dominica, Bangladesh, and Malawi [70], and resilience to disasters is linked to socio-economic status [71]. Accordingly, even if our study does not strictly show a direct causality between disasters, and child poverty, it nevertheless, captures a plausible association between the latter two events.

In a post-2015 policy framework, our paper has some important policy implications. The newly launched Sustainable Development Goals focuses more explicit on the adverse effects of climate change and natural disasters (goal 13), effective institutions (goal 16), and ending global poverty (goal 1). This paper is the first to test the association between elements of these three goals, using both macro and micro data. The paper finds that goal 13 and 16 have a direct association to achieving goal 1. However, it also finds, contrary to what some development researchers would maintain, that most if not all development goals can be reduced to combating corruption, building strong institutions or focus on economic growth (cf. [31, 66, 72]). Environmental mechanisms have a direct impact on peoples´ livelihood regardless of how well-governments are functioning. Hence, combating the negative effects of climate change will require targeted environmental policies and they need to be implemented globally and equitably.

Although long-term global climate change policies as reducing fossil fuel are important [73], our study shows that policy-makers need to counter the immediate effects of climate change to protect vulnerable groups [74]. On the micro-level, we urge governments with a high rate of natural disasters—India, Kenya, Bangladesh, Ethiopia, and Thailand—to devise a social protective net for families: targeting those with several children and younger ones [75]. While anti-poverty cash programs might help in a natural disasters resilience framework (e.g. Bolsa Familia), our analysis suggests that governments need to take into account the various dimensions of poverty: from providing food supplements to building proper housing.

We emphasize that even if this study cannot disentangle the causal direction between the disaster victimization and absolute child poverty, our results show that policy makers in the global arena—such as the United Nations, the International Monetary Fund, or the World Bank—need to pay a special attention to countries that have both high rate of child poverty and a high rate of disaster victimization. It could be that high rate of child poverty also brings about a higher rate of disaster victimization, or vice versa. Whichever way it is—most likely we are facing mutual causation—these children need to be protected.

The Sustainable Development Goal number 13 is a principle declaration to halt climate change, but it lack a clear plan to how the economic and social burden should be shared among the global community [76, 77]. Although more industrialized countries and past generations have enjoyed the fruits of rapid economic development, the real costs on less industrialized, poorer countries, and posterity are becoming painfully apparent. We are letting our and our global neighbors’ children paying these cost with their health and lives. It is time for more equitable policies.
