# Correction: What Is the Association between Absolute Child Poverty, Poor Governance, and Natural Disasters? A Global Comparison of Some of the Realities of Climate Change

**DOI:** 10.1371/journal.pone.0155653

**Published:** 2016-05-10

**Authors:** Adel Daoud, Björn Halleröd, Debarati Guha-Sapir

The affiliation for the second author is incorrect. Björn Halleröd is affiliated with: Department of Sociology and Work Science, University of Gothenburg, Gothenburg, Sweden.

In the Funding section, the grant number from the funder Swedish Research Council is not listed. The grant number is: (dnr: 2011–7619).
